# Does the “Weekend Effect” Extend to Friday Admissions? An Analysis of Ischemic Stroke Hospitalizations in South Carolina

**DOI:** 10.3389/fneur.2020.00424

**Published:** 2020-06-05

**Authors:** Laite Chen, Lu Shi, Donglan Zhang, Chenyang Jiang, Khoa Truong

**Affiliations:** ^1^School of Medicine, Zhejiang University, Hangzhou, China; ^2^Department of Public Health Sciences, Clemson University, Clemson, SC, United States; ^3^Department of Health Policy and Management, College of Public Health, University of Georgia, Athens, GA, United States; ^4^Sir Run Run Shaw Hospital, School of Medicine, Zhejiang University, Hangzhou, China

**Keywords:** temporal pattern of hospital outcomes, discharge to hospice care, ischemic stroke, weekend effect, length of hospital stay

## Abstract

**Background:** Weekend admission has been found to be associated with higher hospital mortality and longer hospital stay among patients with acute cardiovascular conditions. Whether those admitted on Fridays face similar risk as those admitted on Sundays and Saturdays remain uncertain.

**Methods:** This study used 2012–2013 data from hospital records for nonfatal patients with ischemic stroke in the state of South Carolina. The database contained the records of all hospitalizations in South Carolina except military and federal institutions. Multilevel logistic, negative binomial, and log-linear regression models were performed to explore the temporal pattern by hospital admission on specific day of a week for three outcomes: discharge to hospice care (vs. other alive discharges), length of stay, and total charge, respectively. Each model controlled for the patient age, gender, race/ethnicity, year of hospital admission, season of admission, payer, and Charlson Comorbidity Index.

**Results:** A total of 19,346 nonfatal ischemic stroke hospitalizations were identified. Multilevel logistic regression shows that patients admitted on non-Friday weekdays had a significantly lower odds of being discharged to hospice care as compared with those admitted on Fridays [odds ratio = 0.80, 95% confidence interval (CI) = 0.65–0.99] where there was no significant difference between Friday admissions and weekend admissions. The length of hospital stay for admission on non-Friday weekdays was significantly shorter than that of Friday admissions [incident rate ratio (IRR) = 0.87, 95% CI = 0.84–0.90], whereas longer length of hospital stay was found on weekend admission (IRR = 1.06, 95% CI = 1.02–1.11). Friday admissions were not associated with higher charges as compared with non-Friday weekday admissions.

**Conclusions:** Some hospitalization outcomes admitted on Fridays seemed to have the “weekend effects” too. Further studies are warranted to investigate underlying mechanism for such a difference in outcomes between Friday and other weekdays. Interventions to close the weekend–weekday gap of patient outcomes need to consider the elevated risk on Friday admission.

## Introduction

Ample literature has shown that the mortality among patients with severe acute conditions is higher for weekend hospital admission than weekdays (1–11). One common explanation to this phenomenon was that quality of care was compromised because of reduced hospital staffing levels during weekends (12, 13). However, it is not clear whether excess risk started earlier, whereby patients might encounter discontinuity of care due to the transition of clinician teams from Friday onto weekend shifts as well as the possible effect of understaffed weekend care. Most previous studies used the weekend versus weekday dichotomy. Such dichotomization limits the opportunity to detect more specific temporal patterns on transitional days.

It is common in the literature of the “weekend effect” that patients discharged for hospice care were coded as survivors as other alive discharges. Combining discharge to hospice care with other alive discharges would ignore the fact that hospice patients faced a much higher risk of death within a short period. Approximately half of them would die within 3 weeks, and 35.7% of patients die within 1 week (14).

Ischemic stroke is largely responsible for stroke hospitalizations (15). In this study, we aimed to investigate whether the weekend effect started earlier by comparing ischemic stroke patients admitted on Fridays versus patients admitted on non-Friday weekdays and weekends for three hospital outcomes: discharge to hospice care versus other alive discharges, length of stay, and total hospital charges. If these possible temporal patterns exist, it is important to detect them in the efforts of improving patient outcomes. We limit our analysis to nonfatal ischemic stroke hospitalizations, because mortality could shorten the hospitalization and reduce the total charges.

## Data and Statistical Methods

The data for this study came from the hospital records from 2012 to 2013 for all patients with ischemic stroke in the state of South Carolina (16). The database contained information on every hospitalization in all hospitals in South Carolina, excepting military and federal institutions. Patients' demographic characteristics, diagnostic codes, day of hospital admission, discharge disposition, and hospital charges were linked beforehand to the death records by the State Office of Research and Statistics, with personal and hospital identifiers stripped (17). Patients discharged with a primary diagnosis of ischemic stroke were identified based on the following *International Classification of Diseases, Ninth Revision, Clinical Modification* codes: 433.x1, 434.91, 434.11, 434.01, and 436.1 (17). This study was approved by the institutional review board of Clemson University.

The first dependent variable was binary: discharge to hospice care (at home or medical facility) versus other types of nonfatal discharge. The other two outcomes were continuous: length of stay (number of days) and total hospital charges (in dollars).

The key independent variables were two dummy variables based on admission days: weekends (Saturday, Sunday) and non-Friday weekdays (Monday, Tuesday, Wednesday, Thursday), whereas Friday served as the reference group.

Seasonal disparity has been found in the neurological deficits that could impact the outcome of patients with ischemic stroke (18, 19). Therefore, the season of admission was also considered in our analyses: December, January, and February were coded as winter; March, April, and May coded as spring; June, July, and August coded as summer; whereas September, October, and November were coded as fall (20). Summer was set as the reference category because it had been documented as having the lowest stroke mortality of all seasons (21). Patient characteristics controlled in the regression models included age, gender, and race. Given the number of nonwhite nonblack ischemic stroke patients in South Carolina was not significant, they were combined with black patients. Race was therefore categorized as non-Hispanic white versus other races. The models also controlled for the year of hospital admission (2012 vs. 2013), medical cost payers (self-pay vs. Medicare, Medicaid, commercial insurance, worker's compensation, other government, and health maintenance organization), and Charlson Comorbidity Index (CCI) score (0 vs. 1, 2, >2) (22, 23).

To account for clustering of patients within each hospital site, multilevel modeling with random intercept was used. We ran multilevel logistic regression for the binary outcome of discharge status, multilevel negative binomial regression for the length of stay, and the multilevel log-linear for the total charges. All statistical analyses were performed with Stata 12 (Stata Corp., College Station, TX, USA).

## Results

Table 1 provides information on the discharge to hospice care and patient characteristics. Of 19,346 nonfatal ischemic stroke hospitalizations identified for our analysis, 4.08% were discharged to hospice care. This rate was notably higher on Sunday (5.63%) and Saturday (5.22%) as compared with weekdays of which Friday had the highest rate (4.67%). A similar pattern was observed for the CCI; the average CCI was highest on Saturday (2.01) and Sunday (1.99). Meanwhile, the number of ischemic stroke admissions was lowest on Saturday (2,079) and Sunday (2,048) compared to the peaks on Monday (3,021) and Tuesday (3,292). The percentage of cases female patients was highest on Saturday (54.70%) and lowest on Thursday (47.77%), whereas no statistical difference in age was found across admission days.

**Table 1:** Charlson Comordibity Index, and discharge to hospice by the admission day in a week among ischemic stroke patients in South Carolina 2012-2013.

**Admission day**	**Count of ischemic stroke hospitalizations**	**Discharged to hospice among nonfatal discharges**	**Average Charlson Comorbidity Index**	**Sex (%female)**	**Age**
Sunday	2,079	5.63%	1.99	51.52%	63.73
Monday	3,201	3.34%	1.80	49.69%	64.28
Tuesday	3,292	3.07%	1.81	50.83%	65.02
Wednesday	3,067	3.72%	1.82	51.85%	64.57
Thursday	2,964	3.98%	1.86	47.77%	64.59
Friday	2,700	4.67%	1.86	50.95%	63.64
Saturday	2,048	5.22%	2.01	54.70%	64.85
*N*	19346	19346	19346	19346	19346
*P value*	<0.0001	0.11	<0.0001	<0.0001	0.86

Table 2 provides the descriptive statistics of the nonfatal patients by admission day for four dimensions: season, year, race, and primary payor. Summer has the lowest percentage of cases (23.53%), but that is not necessarily true for all days of a week. Summer had the second highest percentage of cases during Sunday (26.52%) compared to Sunday of the other seasons. Friday admission was highest during the fall (28.8%) and lowest during the winter (21.52%). Most of the nonfatal ischemic stroke inpatients were non-Hispanic whites (58.22%). Medicare was the largest payor (52.18%), followed by commercial insurance (20.36%).

**Table 2:** Descriptive statistics for the nonfatal ischemic stroke inpatient cases in South Carolina, 2012-2013.

	**Admission Day of the Week**							
**Season**	Sunday	Monday	Tuesday	Wednesday	Thursday	Friday	Saturday	Total
Winter	27.27	23.75	22.92	23.57	25.09	21.52	25.84	24.2
Spring	21.21	28.44	27.24	25.93	24.4	27.53	27.52	26.16
Summer	26.52	23.13	24.92	23.23	20.27	22.15	24.83	23.53
Fall	25	24.69	24.92	27.27	30.24	28.8	21.81	26.11
**Year**								
2011	1.14	1.88	1.66	0.34	1.37	1.27	1.68	1.34
2012	53.41	44.69	48.84	45.12	48.11	49.68	47.65	48.11
2013	45.45	53.44	49.5	54.55	50.52	49.05	50.67	50.55
**Race**								
Nonwhite	42.42	40	41.53	37.71	42.61	41.14	47.32	41.78
White	57.58	60	58.47	62.29	57.39	58.86	52.68	58.22
**Primary payor**								
Self-pay	13.26	11.56	12.62	10.1	11	12.03	14.43	12.12
Medicare	54.92	52.81	53.82	49.83	54.64	48.42	51.34	52.18
Medicaid	4.92	6.25	6.64	5.72	7.56	7.28	6.04	6.37
Commercil	17.05	21.56	18.27	24.58	19.59	20.89	20.13	20.36
Government and charitable	1.52	1.56	0.33	2.36	1.72	1.9	1.34	1.53
Other	8.33	6.25	8.31	7.41	5.5	9.49	6.71	7.43

Multilevel models' results are presented in Table 3 where Friday admission served as the reference group. After adjusting for patient characteristics and other controlling variables, ischemic stroke patients admitted on non-Friday weekdays were less likely to be discharged to hospice care as compared with those admitted on Friday [odds ratio (OR) = 0.80, 95% confidence interval (CI) = 0.65,0.99], whereas weekend admissions were not significantly different from Friday admissions in the likelihood of being discharged to hospice care (OR = 1.12, 95% CI = 0.88–1.42).

**Table 3:** Multilevel models of nonfatal outcomes and “Friday effect” among ischemic stroke patients in South Carolina, 2012-2013.

**Admitted on weekend**	**Discharged to hospice**	**Length of hospital stay**	**Total charge**
**(Friday as reference)**	**Odds ratios**^**1**^	**Incident Rate Ratios**^**2**^	**β** **coefficient**^**3**^
Non-Friday weekdays	0.80^**^ [0.65,0.99]	0.87^***^ [0.84, 0.90]	−560.92 [-2264.50, 1121.66]
Weekends	1.12 [0.88, 1.42]	1.06^**^ [1.02, 1.11]	708.32 [-1251.71, 2668.36]
*N*	19346	19346	19346

*We use multilevel logistic regression for the discharge to hospice outcome*.

*We use multilevel negative binomial model for the length of stay outcome*.

*We use multilevel loglinear model for the total charge outcome*.

*We also control for age, gender, race/ethnicity, payer status, season of admission and admission year in these four random-effects multilevel logistic regressions, with hospital as the cluster variable*.

*95% confidence intervals in parentheses*.

*p < 0.05*,

** *p < 0.01*,

*** *p < 0.001*.

Similar pattern was found on the length of hospital stay. Non-Friday admissions' length of stay was a little shorter than Friday admissions' (incident rate ratio (IRR) = 0.87, 95% CI = 0.84–0.90). On the contrary, patients admitted on weekends had a prolonged length of hospital stay (IRR = 1.06, 95% CI = 1.02–1.11).

For the third outcome, total charges, the results were not significantly different, whereas sign of the coefficient was as expected: negative for non-Friday weekdays and positive for weekends.

## Discussion

Using ischemic stroke inpatients' data in the entire state of South Carolina during 2012–2013, we found that patients admitted on weekends and Fridays were more likely to be discharged to hospice care and had longer length of stay compared with patients admitted on non-Friday weekdays. Before coming to any conclusion that the “weekend effect” could start as early as Friday, we discuss a few possible competing explanations for our findings. First, one needs to consider the possibility that patients admitted on non-Friday weekday had worse outcome than those admitted on Friday and weekend as they were more likely to die during their hospitalization, thus cutting the overall likelihood of hospice care. We therefore conducted an extra analysis of mortality risk for ischemic stroke inpatients by adding fatal outcomes to our analytic sample (because the results above included only nonfatal discharge cases). Table 4 presents the mortality rate among all discharges by admission day. As shown, admission from Monday through Thursday has a lower mortality risk (<4% for each day) compared to that of Friday admission (4.42%), Saturday admission (5.8%), and Sunday admission (4.33%). These statistics seem to provide a reasonable argument that non-Friday admission did not have a worse outcome than Friday and weekend admission using fatal outcome as a measure.

**Table 4A:** Mortality risk by admission day among ischemic stroke patients in South Carolina 2012-2013.

**Admission day**	**Mortality among all discharges**
Sunday	4.33%
Monday	3.99%
Tuesday	3.66%
Wednesday	3.80%
Thursday	3.67%
Friday	4.42%
Saturday	5.80%

**Table 4B:** Frequency of types of discharge status.

**Outcomes**	**Sunday**	**Monday**	**Tuesday**	**Wednesday**	**Thursday**	**Friday**	**Saturday**	**Total**
Alive, non-hospice	94.86	96.19	96.32	96.15	96.18	95.91	94.90	95.91
Expired	2.40	1.86	1.78	1.86	1.82	1.90	2.33	1.94
Discharge to hospice	2.74	1.95	1.91	1.99	2.00	2.19	2.78	2.15

Second, one can argue that there is no difference in care and mortality risk by day of admission, but care providers deliberately discharged patients earlier (to hospice care) to free up beds. Although it is not possible to rule out this possibility completely because of the lack of such information on our data set, it is not entirely clear how admission day could be linked to discharge decision to reduce caseload or free up beds as there is a lot of dynamics going on between total length of stay, patient volume, and staff size at any point in time or day of a week. Our finding also suggests that patients admitted on weekdays had shorter length of stay, but there was no indication that discharge day was more likely to fall on Fridays or weekends.

Third, there is a potential biased estimation of the Friday or “weekend effect” as admission day is confounded with unobserved patient health status. A typical “story” for the third variable problem is that those admitted on Fridays or weekends were sicker, thus making them more likely to be discharged to hospice care, and they somehow were more likely to be brought to hospital on Fridays or weekends. Given the nature of a cross-sectional study, we tried to reduce this problem by controlling for the CCI.

Last but not least, patients admitted on a Friday were sicker and more likely to die and be discharged to hospice care compared to those admitted on a non-Friday weekend. It is a typical self-section problem where one cannot conclude that quality of hospital care would be responsible for the discharge outcome. This challenge is similar to the previous one in an observational study, and we therefore need to be conservative in our conclusion about extended weekend effect.

Having all the above discussed, our findings do show that Friday admissions were more likely to result in hospice care and longer length of stay compared to non-Friday admissions. Some previous studies have shown that weekend admissions had compromised quality issues such as a longer wait time for procedures (24) and a lower likelihood to get prompt intervention in time (25, 26). Possible explanation for shortfalls in quality of medical care includes staff shifting between weekday and weekend staff (7, 27) or the understaffed weekend shift that immediately follows a Friday admission (28).

Several studies have identified hospital-level predictors of “weekend effect” in the adverse outcomes. A study in Florida identified that full adoption of electronic medical records, home health program, pain management program, increased registered nurse–to–bed ratio, and inpatient physical rehabilitation might overcome the “weekend effect” on the patient's length of stay in the hospital (29, 30). Another study in New Jersey of patients with a primary diagnosis of cerebral infarction found no difference between weekend admissions and weekday admissions in 90-day mortality for patients admitted to comprehensive stroke centers, while the excess mortality among weekend admissions persisted for patients admitted to other health care organizations (31). A study from France found that the deleterious effect of weekends/holidays on early stroke mortality disappeared after the implementation of a dedicated stroke care network with 24/7 availability of a stroke-trained physician team (32). In addition, interhospital transfer is critical to condition management, suggesting the evaluation of weekend effect may be subject to clinical characteristics (28, 33). A study of the transfers to the intensive care unit throughout the week (34) has demonstrated that Sunday (and trend toward Friday) had a higher 30-day mortality, as compared with weekday transfer, suggesting worse outcomes were associated with off-peak times of transfer.

Limitations of this study should be noted while interpreting our results. First, the analysis cannot fully rule out the possibility that conditions of patients admitted on Fridays were more severe than those admitted on non-Friday weekdays as discussed above. Some have argued that the CCI may not adequately reflect the severity of disease among ischemic stroke patients (35). Second, the patient end points at discharge may also be sensitive to their admission vital signs including weight, systolic blood pressure, and laboratory values (36), which are not available from hospital discharge data. Third, only three outcomes were included in our analysis, whereas other measures such as postdischarge mortality, readmission, and quality of life would be important to consider too (37). Last but not least, hospital transfer was not available in our data; hence, the attribution to the medical charge was not discussed in the present study.

While more studies are warranted to examine and explain this association, providers with more staffing and infrastructure resources such as stroke network and comprehensive stroke centers have been shown as more effective in avoiding this weekend effect (38–40). Efforts to close the weekend–weekday outcome gap might need to consider Friday admissions.

## Data Availability Statement

The datasets analyzed in this articlw are not publicly available. Requests to access the datasets should be directed to lus@clemson.edu.

## Ethics Statement

This study was approved by the Institutional Review Board of Clemson University.

## Author Contributions

CJ and LS: conceptualization and validation. LC and LS: methodology and formal analysis. LS and DZ: software. DZ and KT: investigation. LS: truong, resources and project administration. DZ: data curation. LC: writing-original draft preparation. LS, DZ and KT: writing-review and editing. CJ: truong, supervision.

## Conflict of Interest

The authors declare that the research was conducted in the absence of any commercial or financial relationships that could be construed as a potential conflict of interest.
